# Application of Response Surface Methodology (RSM) for the Optimization of Ultrasound-Assisted Extraction (UAE) of *Moringa oleifera*: Extraction Yield, Content of Bioactive Compounds, and Biological Effects In Vitro

**DOI:** 10.3390/plants12132455

**Published:** 2023-06-26

**Authors:** Wahyuning Setyani, Retno Murwanti, Teuku Nanda Saifullah Sulaiman, Triana Hertiani

**Affiliations:** 1 Pharmaceutical Sciences Doctoral Study Program, Faculty of Pharmacy, Universitas Gadjah Mada, Yogyakarta 55281, Indonesia; 2 Department of Pharmaceutics and Pharmaceutical Technology, Faculty of Pharmacy, Universitas Sanata Dharma, Yogyakarta 55282, Indonesia; 3 Department of Pharmacology and Clinical Pharmacy, Faculty of Pharmacy, Universitas Gadjah Mada, Yogyakarta 55281, Indonesia; 4 Department of Pharmaceutical Technology, Faculty of Pharmacy, Universitas Gadjah Mada, Yogyakarta 55281, Indonesia; 5 Department of Pharmaceutical Biology, Faculty of Pharmacy, Universitas Gadjah Mada, Yogyakarta 55281, Indonesia

**Keywords:** bioactive compounds, Box–Behnken design, *M. oleifera*, response surface method, ultrasound-assisted extraction

## Abstract

This study optimized ultrasound-assisted extraction conditions to maximize the extraction yield, total flavonoid content (TFC), total phenolic content (TPC), and DPP IV enzyme inhibitory activity from *Moringa oleifera*. The four UAE factors, solvent ratio (A), solvent–solid ratio (B), extraction temperature (C), and extraction time (D), were optimized using response surface methodology (RSM). A Box–Behnken design was used for the experimental design. The optimal conditions were found to be a 50% *v/v* solvent ratio, a 30% *v/w* solvent–solid ratio, 35 °C extraction temperature, and 45 min extraction time. The experimental value of extraction yield (*R*_1_), TFC (*R*_2_), TPC (*R*_3_), and DPP IV enzyme inhibitory activity (*R*_4_) (87.99% *w/w*, 56.63 mg QE/g extract, 97.26 mg GAE/g extract, and 93.32% inhibition, respectively) agreed with those predicted by RSM models (88.10% *w/w*, 56.61 mg QE/g extract, 97.16 mg GAE/g extract, and93.38% inhibition, respectively), thus demonstrating the appropriateness of the model used and the ability of the RSM to optimize the extraction conditions. Excellent DPP IV enzyme inhibitory activity was exhibited by *M. oleifera* compared with the standard, sitagliptin. While the modeled equation fits the data, the t-test is not significant, suggesting that the experimental values agree with those predicted by the RSM–BBD

## 1. Introduction

A key source of bioactive compounds for developing new anti-diabetic medications for insulin resistance is now natural products. Recently, more and more natural components have been described as having antidiabetic properties in insulin resistance, and many attempts have been made to elucidate the mechanisms by which these occur [1]. Recent research has shown that a variety of flavonoids have antidiabetic properties, including promoting the development of diabetes and its consequences by altering the lipid profile, glucose metabolism, and liver enzyme activity [2]. The valuable bioactive compounds in *M. oleifera* leaves are myricetin, quercetin, and kaempferol, which are present in concentrations of 5.8, 0.207, and 7.57 mg/g, respectively [3,4]. In dried leaves, gallic acid is the most abundant, with a concentration of 1.034 mg/g of dry weight. The concentration of chlorogenic and caffeic acids range from 0.018 to 0.489 mg/g of dry weight and 0.409 mg/g of dry weight, respectively [5,6]. *M. oleifera* leaf extract is a potential instrument for insulin resistance in diabetes mellitus. The presence of the flavonoid activity of Moringa leaves (*M. oleifera*) encouraged the development of optimized extraction methods. To achieve this, it is necessary to choose the right method in the extraction process to obtain an optimal compound content. When analyzing the chemical components present in a plant, the choice of extraction process must be taken into account [7]. In general, it is feasible to obtain chemical compounds of high value, such as flavonoids, which are known to have biologically important qualities, by understanding the most effective extraction conditions [8,9].

There are two extraction techniques, namely conventional and non-conventional. In conventional extraction, compounds are extracted from the plant matrix, employing diffusion and mass transfer principles after the plant matrix has been homogenized and submerged in one or more solvents. Although this approach is fairly straightforward, it has disadvantages, such as low efficiency because of the high solvent requirements [10]. At the same time, compared with standard procedures, non-conventional extraction requires specific equipment and a certain amount of energy to boost efficiency or selectivity [11]. Extraction is an important means of identifying, isolating, and applying valuable bioactive compounds from natural plants. One of the most feasible non-conventional extraction techniques for obtaining bioactive substances from specific plant components, such as flowers, fruit, leaves, bark, seeds, and pods, is ultrasound-assisted extraction (UAE) [12,13]. Based on the cavitation produced in the material, UAE can boost the extraction mass-transfer rate. Increased release of bioactive chemicals from plant materials into the liquid phase of extraction occurs due to the destruction of the polymer wall structure. UAE permits a quick extraction period since the analyte is more soluble in the extraction media [14,15,16].

One of the aims of this research is to optimize the extraction method of UAE using the Response Surface Methodology (RSM) approach. BBD is a type of RSM-independent design that is recommended for optimization processes with three or more independent variables [17]. Box–Behnken Design (Design-Expert Software@ version 12.0, Stat-Ease Inc., Minneapolis, MN, USA) was used as the model fitting design software. Several studies have previously been carried out to test the inhibitory activity of the DPP IV enzyme, one of the dependent variables in this study [17,18,19], including optimizing the UAE extraction method with RSM–BBD. In another study, the TFC and TPC were determined [20,21]. The TFC was determined from the regression equation of the calibration curve (y = 0.6942x − 0.0042, *R*^2^ = 0.99) and is expressed in QE, varying between 51.86–77.00 mg/g in QE. The TPC was determined from the regression equation of the calibration curve (y = 0.0018x − 0.0469, *R*^2^= 0.99) and is expressed in GAE, varying between 70.06–100.98 mg/g in GAE [22]. Research on docking studies, the synthesis of compounds and their analogues has also been carried out [23]

Several studies have previously been conducted for the extraction and quantification of *M. oleifera* constituents. However, the application of response surface methodology (RSM) for the optimization of ultrasound-assisted extraction (UAE) of *Moringa oleifera* with respect to the extraction yield, the content of bioactive compounds, and biological effects in vitro has not previously been undertaken. On this basis, we aimed to study the effects of different UAE extraction variables (solvent ratio, solvent–solid ratio, temperature, and time) on extraction yield (*R*_1_), TFC (*R*_2_), TPC (*R*_3_), and DPP IV enzyme inhibitory activity (*R*_4_).

## 2. Results and Discussion

### 2.1. BBD Method Optimization of Extraction Conditions

The ranges for the four independent extraction variables, namely, solvent ratio (A), solvent–solid ratio (B), temperature (C), and time (D), at levels (−1, 0, +1) for extraction parameter optimization by the BBD method were selected based on observations from the single-factor experiments [24] (Table 1).

### 2.2. Statistical Analysis and Model Fitting

All independent variables were employed with the BBD to investigate the effects on the extraction yield, TFC, TPC, and DPP IV enzyme inhibitory activity in *M. oleifera*. In Table 2, the results of 27 experimental combinations of four independent extraction variables were recorded to investigate their impact on *R*_1_, *R*_2_, *R*_3_, and *R*_4_. The results were fitted into Equation (1) (i.e., a second-order polynomial equation):

(1) $$Y=\beta_{0}+\overset{4}{\underset{i=1}{\sum }}\beta_{i}X_{i}+\overset{4}{\underset{i=1}{\sum }}\beta_{ii}X_{i}^{2}+\overset{3}{\underset{i=1}{\sum }}\overset{4}{\underset{i<j}{\sum }}\beta_{ij}X_{i}X_{j}$$

where *Y* is the expected matter of *D*, *β_0_* is the intersection of the equations, *β_i_* is the linear coefficient, *β_ii_* is the squared coefficient, *β_ij_* is the coefficient of the cross outcome, and *X_i_* and *X_j_* are the coded independent variables. The F-test is used to determine a polynomial equation’s relevance. Each coefficient in the polynomial equation’s statistical significance is evaluated using the *p*-value [24,25] to generate the following equations with coded factors for dependent variables (*R*_1_–*R*_5_):

Table 3 presents the design matrices of the experiments using the BBD and the predicted data for the response variables. The following are the actual values of the response variables: extraction yield (72.71–94.20 *w*/*w*), TFC (51.86–77.00 mg QE/g extract), TPC (70.06–101.48 mg GAE/g extract), and DPP IV enzyme inhibitory activity (42.86–90.42% inhibition).

For the BBD-based experimental design, a quadratic model with an *R*^2^ of 0.9691, 0.9864, 0.9317, and 0.9323 was found to be the best-fit models for the analysis of *R*_1_, *R*_2_, *R*_3_, and *R*_4_, respectively. In Table 4 and Table 5, the regression analysis and response regression equation data for the suggested model are listed for *R*_1_, *R*_2_, *R*_3_, and *R*_4_.

The adjusted *R*^2^/predicted *R*^2^ values for *R*_1_, *R*_2_, *R*_3_, and *R*_4_ (0.9165/0.8218, 0.9540/0.8681, 0.9054/0.7843, 0.9134/0.7079, respectively) were found to be close to showing a correlation between adjusted and predicted values. Furthermore, while differences between squared and predicted *R* values should be less than 0.2 for every dependent variable (indicating the fit for measuring whether the precisions are equal), the signal-to-noise ratio can be used, which should be greater than 4 to fit the model. In this experiment, the signal-to-noise ratio for *R*_1_, *R*_2_, *R*_3_, and *R*_4_ were 7.4695, 8.9156, 8.3776, and 15.8775, respectively; all the values were more than 4, indicating that the models were fitted correctly and suggesting that the proposed model can be used to navigate the design space. The ANOVA (analysis of variance) results for the quadratic models of *R*_1_, *R*_2_, *R*_3_, and *R*_4_ are listed.

The model F-values for yield were 5.69, implying that the deal is significant. There is only a 0.23% chance that an F-value this large occurs due to noise. Similarly, for *R*_2_, *R*_3_, and *R*_4_, the F-values were found to be 6.69, 4.24, and 11.81, respectively, implying that each quadratic model was significant and that there was only a 0.11, 0.83, and 0.01% chance, respectively, that these F-values could occur due the noise. The *p*-values for the proposed quadratic model of all dependent variables were found to be very low (<0.05), suggesting that the models developed for the analysis of all the variables were significant. Lack of fit F-values of 1.02, 0.45, 0.27, and 0.29 for *R*_1_, *R*_2_, *R*_3_, and *R*_4_, respectively, imply that the lack of fit is not significant relative to the pure error; thus, it is appropriate to fit the model and predict the response. There is a 59.30, 83.97, 93.96, and 92.91% chance that a lack of fit F-values this large could occur due to noise for the variable yields, TFC, TPC, and DPP IV enzyme inhibitory activity, respectively.

### 2.3. Effect of Independent Variables (A, B, C, and D) of Ultrasound-Assisted Extraction on R_1_, R_2_, R_3_, and R_4_

The influences of the individual independent variables A, B, C, and D are listed in Table 4. The quadratic effects of four variables were found to be significant (*p* < 0.05) and to affect *R*_1_, *R*_2_, *R*_3_, and *R*_4._ The quadratic effect of the solvent ratio (A) exhibited significance and affected *R*_4_. The effect on the solvent–solid ratio (B) indicated significance and affected *R*_2_ and *R*_4._ Temperature (C) showed a significant effect on *R*_1_ and *R*_4._ Time (D) exhibited a significant effect and affected only *R*_3_.

### 2.4. Response Surface Analysis of R_1_, R_2_, R_3_, and R_4_

**Figure 1:**
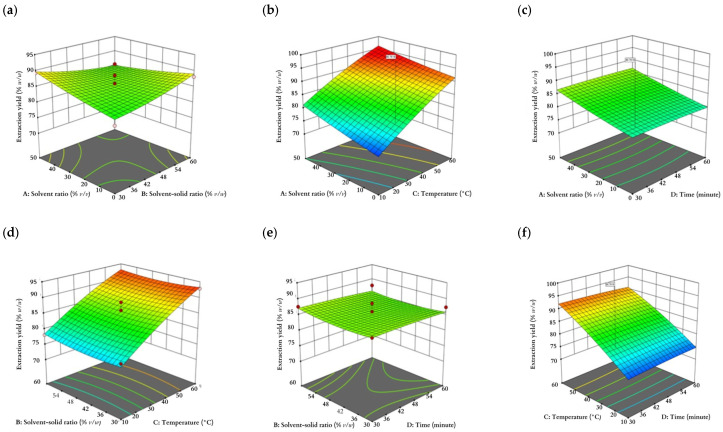

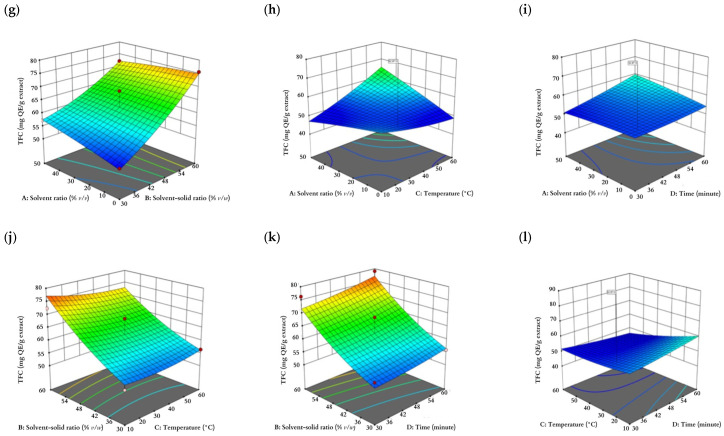
Response surface plots showing (**a**–**f**) the interaction effect of extraction yield as a function of solvent ratio, solvent–solid ratio, temperature, and time and (**g**–**l**) the interaction effect of TFC as a function of solvent ratio, solvent–solid ratio, temperature, and time.

**Figure 2:**
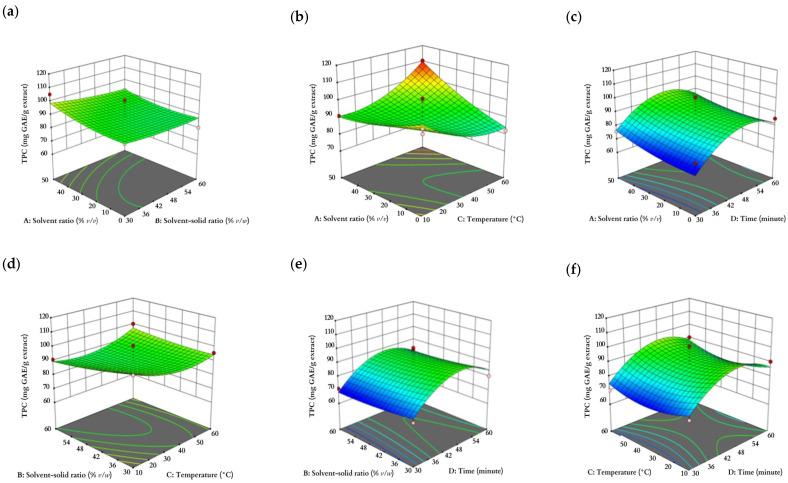

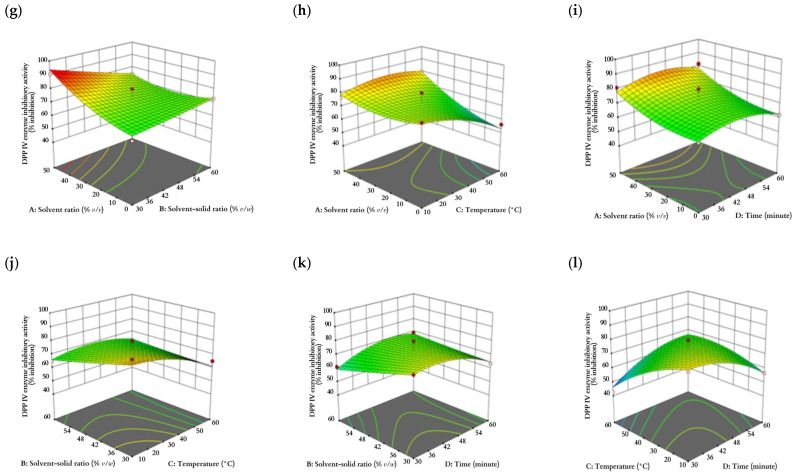
Response surface plots showing (**a**–**f**) the interaction effect of TPC as a function of solvent ratio, solvent–solid ratio, temperature, and time and (**g**–**l**) the interaction effect of DPP IV enzyme inhibitory activity as a function of solvent ratio, solvent–solid ratio, temperature, and time.

As shown in Figure 1 and Figure 2**,** the solvent ratio, solvent–solid ratio, temperature, and time were interpreted in the range of (0, 25, 50% *v/v*), (30, 45, 60% *v/w*), (10, 35, 60 °C), and (30, 45, 60 min), respectively. The confidence interval for each response was 95% in the mentioned ranges on the plots.

### 2.5. Verification of the Model

**Figure 3:**
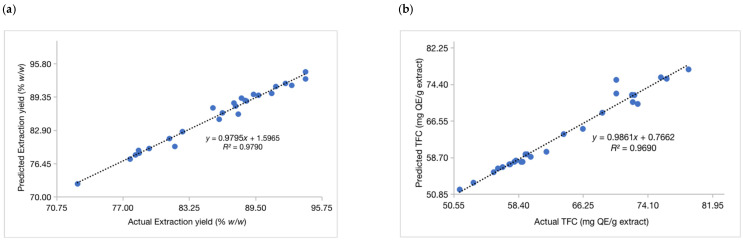

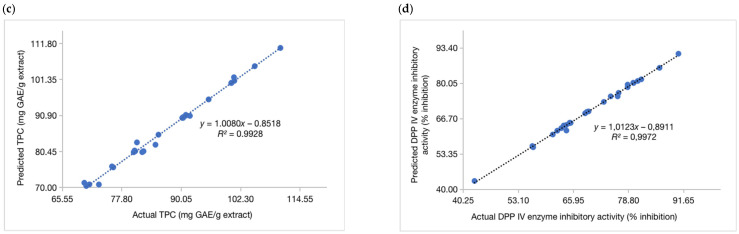
Comparison between predicted and actual values of the response variables an extraction yield (**a**) TFC (**b**) TPC (**c**) DPP IV enzyme inhibitory activity (**d**).

A good fit with high correlation is achieved if the regression model has an *R*^2^ value above 0.9. The R values obtained indicated that more than 99% of the response variables (*R*_1_–*R*_4_) could be described by the RSM model. The high value of *R*^2^ for each response indicated that the BBD design fitted well into the quadratic polynomial models that were developed. These results confirmed the predictability of the models in determining the optimum conditions needed to obtain the highest extraction yield, TFC, TPC, and DPP IV enzyme inhibitory activity (Figure 3).

In order to determine the adequacy of the final model, three randomized validation sets were performed to verify the models (Table 6). The results were compared with predicted values by calculating the RSE percentages. RSE values lower than five were considered to be in agreement with the predicted values. The RSE values obtained indicated no significant differences between the actual and predicted values, proving that the models were adequate.

### 2.6. Optimized Conditions of the Extraction Parameters

Optimized conditions for the simultaneous maximum extraction yield, TFC, TPC, and DPP IV enzyme inhibitory activity were determined. In the BBD analysis, the optimized conditions using the solvent ratio (50% *v/v*), solvent–solid ratio (50% *v/w*), temperature (35°), and time (45 min) could produce the optimum extraction yield, TFC, TPC, and DPP IV enzyme inhibitory activity (87.99% *w/w*, 56.63 mg QE/g extract, 97.26 mg GAE/g extract, and93.32% inhibition, respectively). Table 7 shows the predicted and actual response values for the optimized conditions. Under optimum conditions, the actual responses showed that the models were in good agreement with the predicted values, with RSE values of less than 0.2%.

## 3. Material and Methods

### 3.1. Apparatus and Reagents

The following apparatus were used in this study: Erlenmeyer flasks (Merck, Darmstadt, Germany ), glass beakers (Merck, Darmstadt, Germany), measuring cups (Merck, Darmstadt, Germany), ultrasonicator (24 kHz, 200 W, Hielscher GmbH, Oderstr. 53. D-14513 Teltow, Germany), oven, Whatman filter paper no. 42, rotary vacuum evaporator (Buchi, Flawil, Switzerland), analytical balance (Ohaus, 68 Circular Rd, #02-01, Singapore), micropipettes (Accucare, Bekasi, Indonesia), micropipette tips (Surgitech, Huaian, China), 1.5 and 2 mL microtubes (Gsbio, 07-14 Midview Bldg., Singapore), 96-well microplates (Iwaki, Sumedang, West Java, Indonesia), and a multi-mode microplate reader (Synergi HTX, Santa Clara, CA, USA).

Reagents used were 96% ethanol (Merck, Darmstadt, Germany), 10% aluminum chloride (Merck, Darmstadt, Germany), 1 M sodium acetate (Merck, Darmstadt, Germany), Folin–Ciocalteu reagent (Merck, Darmstadt, Germany), sodium carbonate (Merck, Darmstadt, Germany), quercetin (Sigma-Aldrich, St. Louis, MO, USA), gallic acid (Sigma-Aldrich, St. Louis, MO, USA), dimethyl sulfoxide (DMSO) (Merck, Darmstadt, Germany), and a dipeptidyl peptidase IV (DPP IV) inhibitor screening kit consisting of assay buffer (25 μL), enzymes (100 μL), substrate (200 μL) and sitagliptin (50 μL) (Sigma-Aldrich, St. Louis, MO, USA).

### 3.2. Plant Material

The material employed in this study was dried simplicia of Moringa leaves (*M. oleifera*) obtained from the Research Institute for Medicinal Plants and Spices (Balittro), Bogor, Indonesia. The age of the harvested Moringa leaves (*M. oleifera*) was not lower than 30 days. The wet sorting process was carried out manually by separating impurities or other foreign materials prior to the washing process by removing unnecessary parts. The Moringa leaves were dried in an oven at 40 °C until a moisture content of less than 10% was obtained. Dry sorting was performed manually by separating foreign objects such as plant parts that were not needed and other impurities that were still present and left in the dried simplicia. The dried sample was powdered to a particle size of 40 mesh. The drying and grinding procedures were performed for one batch of samples, and these procedures were not replicated.

### 3.3. Ultrasound-Assisted Extraction of M. oleifera leaves

In general, chemical compounds such as flavonoids, which are known to have biologically relevant properties [8,9], can be obtained by understanding the most productive extraction conditions. The extraction method used in this study is the UAE non-conventional extraction method [25,26,27], which tries to improve selectivity or efficiency in comparison with traditional methods [11]. The experimental design approach was carried out by optimizing the extraction conditions [28]. Dried leaves of *M. oleifera* were extracted with the ultrasonic processor UP 200S (24 kHz, 200 W, Hielscher GmbH, Oderstr. 53. D-14513 Teltow, Germany ) using the UAE method, which was preceded by immersion of the sample in a solvent at a certain solvent ratio. The extraction time is calculated when the temperature for each run reaches a predetermined temperature. The extract obtained was filtered using a Whatman^®^ brand membrane filter holder (300 mL capacity) Cat. No. 1960-004, then the filtrate was concentrated with a rotary evaporator until a thick extract was obtained. The percentage yield of the extract was calculated after obtaining a constant viscous extract weight to the weight of the dry powder of *M. oleifera* by using the following equation:

(2) $$\mathrm{Extraction}\;\mathrm{yield}\;\left(\%\right)=\frac{\mathrm{weight}\;\mathrm{of}\;\mathrm{extract}}{\mathrm{weight}\;\mathrm{of}\;\mathrm{dried}\;\mathrm{leaves}}\times 100\%$$

### 3.4. Determination of Total Flavonoid Content (TFC)

A multi-mode microplate reader was used to measure the total flavonoid content of the *M. oleifera* extract. Quercetin was utilized as a reference substance. A 10 µL volume of 10% aluminum chloride was measured and put into a 1.5 mL microtube, to which was added 50 µL of each of the 27 samples of 1 mg/mL thick extract of *M. oleifera* leaves, 150 µL of 96% ethanol, and 10 µL of sodium acetate 1 M. Each of these mixtures was put into a 96-well microplate and shaken in a multi-mode microplate reader for 1 min. These were then incubated at 37 °C protected from light for 40 min and the absorbance was read on a multi-mode microplate reader at λ 415 nm [29]. Total flavonoid compound content was calculated as the mean ± standard deviation (SD) and expressed in mg quercetin equivalent (QE)/g extract [20,30].

### 3.5. Determination of Total Phenolic Content (TPC)

With a multi-mode microplate reader and the Folin–Ciocalteu method, the total phenolic content of *M. oleifera* extract was measured. Gallic acid was employed as a reference ingredient. The thick extract of *M. oleifera* leaves of 1 mg/mL from 27 samples was measured by putting as much as 12.5 µL each into a 96-well microplate, to which was added 50 µL of Folin–Ciocalteu reagent. Then, the microplates were placed in the shaker for 1 min in a multi-mode microplate reader and incubated for 4 min protected from light. A volume of 37.5 µL Na_2_CO_3_ was added, and the samples were shaken for 1 min, incubated for 2 h protected from light, and their absorbance read on a multi-mode microplate reader at λ 765 nm [18]. The total phenolic compound content was calculated as the mean ± standard deviation (SD) and expressed as mg gallic acid equivalent (GAE)/g extract [20,30].

### 3.6. Insulin-Resistant Antidiabetic Assay of BBD-Run M. oleifera Extract Samples

The inhibitory activity of the DPP IV enzyme was determined in vitro using a DPP IV inhibitor screening kit and a multi-mode microplate reader for the 5 test groups, with each group consisting of 3 wells on a 96-well multi-plate. The group divisions were as follows: Group I was a positive control consisting of DPP IV assay buffer (49 µL) and DPP IV enzyme (1 µL) that was shaker-treated for 1 min and incubated for 10 min at 37 °C protected from light, to which was then added DPP IV assay buffer (23 µL) and DPP IV substrate (2 µL). This was shaken for 1 min, and, finally, sitagliptin (2.5 µL dissolved in 22.5 µL DPP IV assay buffer) was added. Group II was the negative control consisting of DPP IV assay buffer (49 µL), DPP IV enzyme (1 µL) that was treated with a shaker for 1 min and incubated for 10 min at 37 °C protected from light, to which was then added DPP IV assay buffer (23 µL) and DPP IV substrate (2 µL). Group III was a blank with DMSO consisting of DPP IV assay buffer (49 µL) that was treated with a shaker for 1 min and incubated for 10 min at 37 °C protected from light, to which was then added DPP IV assay buffer (23 µL) and DPP IV substrate (2 µL). DMSO (1 µL) was added and given a shaker treatment for 1 min and incubated for 10 min at 37 °C protected from light. Group IV was a blank without DMSO consisting of DPP IV assay buffer (49 µL) that was treated with a shaker for 1 min and incubated for 10 min at 37 °C protected from light, to which was then added DPP IV assay buffer (23 µL) and DPP IV substrate (2µL). Group V was a concentrated extract of *M. oleifera* leaves consisting of DPP IV assay buffer (49 µL), DPP IV enzyme (1 µL) that was given a shaker treatment for 1 min and incubated for 10 min at 37 °C protected from light, to which was then added DPP IV assay buffer (23 µL) and DPP IV substrate (2 µL) and the mixture shaken for 1 min. To each of the 27 samples (25 µL) was added the viscous extract that had been dissolved in DPP IV assay buffer until each mixture had a content of 1000 ppm/well [31]. Then the sample was put into the multi-mode microplate reader and the fluorescence (FLU, λex = 360 nm/λem = 460 nm) measured every minute for 15–30 min at 37 °C protected from light during the measurement process. DPP IV enzyme inhibition activity was calculated using the formula:

(3) $$\%\;\mathrm{of}\;\mathrm{DPP}\;\mathrm{IV}\;\mathrm{enzyme}\;\mathrm{inhibitory}\;\mathrm{activity}=\left(\frac{\mathrm{Slope}\;\mathrm{control}\;\mathrm{enzyme}\;-\;\mathrm{Slope}\;\mathrm{sample}\;\mathrm{inhibitor}}{\mathrm{Slope}\;\mathrm{control}\;\mathrm{enzyme}}\right)\times 100$$

### 3.7. BBD Experimental Design

#### 3.7.1. Single-Factor Experimental Design

The extraction variables (solvent ratio, solvent–solid ratio, temperature, and time) were selected based on the observation of single-factor effects, which were used to optimize all extraction variables using the BBD method to obtain maximum values for the total extraction yield, TFC, TPC, and the insulin-resistant antidiabetic activity assay. The impact of single factors on the total extraction yield, TFC, TPC, and the insulin-resistant antidiabetic activity assay was evaluated by varying the solvent ratio (0, 25, and 50% *v/v*), solvent–solid ratio (30, 45, and 60% *v/w*), temperature (10, 35, and 60 °C), and time (30, 45, and 60 min).

#### 3.7.2. Optimization of Extraction Variables Using the BBD Method and Method Validity Testing

A 4-factorial (4^3^) Box–Behnken design (version 12, Design-Expert Software, Stat-Ease Inc., Minneapolis, MN, USA) was used for the optimization of the independent variables solvent ratio (A), solvent–solid ratio (B), temperature (C), and time (D), at low (−1), medium (0), and high levels (+1) (Table 1). A total of 27 experimental runs, comprising four central points, were generated and fitted to a second-order polynomial equation to obtain the yield (*R*_1_), TFC (*R*_2_), TPC (*R*_3_), and DPP IV enzyme inhibitory activity (*R*_4_). Three-dimensional contour plots were made to deduce the independent variable’s effects on *R*_1_, *R*_2_, *R*_3_, and *R*_4_, and the “biggest-is-best” principle was used for each variable to obtain the optimum outcome, with *p*-values ≤ 0.05 considered to be significant. A final confirmation experiment (*n* = 4) was performed using optimized values for model validation.

## 4. Conclusions

This study demonstrated that RSM is an effective tool for optimizing the conditions of *M. oleifera* leaf extraction and allows a better understanding of the relationship between independent variables and response variables. The model was verified statistically with ANOVA. Under the optimal conditions, the actual values were in good agreement with the predicted values, as the RSE values for the optimum conditions were less than 0.2%. The quadratic effect of the solvent ratio (A) exhibited significance and affected *R*_4_. The solvent–solid ratio (B) exhibited significant effects on *R*_2_ and *R*_4._ Temperature (C) exhibited significant effects and affected *R*_1_ and *R*_4._ Time (D) exhibited a significant effect on *R*_3_ and *R*_4._ DPP IV enzyme inhibitory activity was influenced by all independent variables (*p* < 0.05). The optimization conditions were as follows: solvent ratio of50% *v/v*, solvent–solid ratio of30% *v/w*, temperature 35 °C, and time 45 min. Under these conditions, the experimental and model predicted yield value, TFC, TPC, and DPP IV enzyme inhibitory activity (87.99 and 88.10% *w/w*, 56.63 and 56.61 mg QE/g extract, 97.26 mg and 97.16 mg GAE/g extract, and 93.32 and 93.38% inhibition, respectively). Therefore, in this study, *M. oleifera* leaf extraction was successfully optimized.

## Figures and Tables

**Table 1:** Coded independent variables used in the BBD design.

Independent Variable	Factor Level			Dependent Variable			
	−1	0	+1				
A	0	25	50	Extraction yield (% *w/w*) (*R*_1_)	TFC (mg QE/g extract) (*R*_2_)	TPC (mg GAE/g extract) (*R*_3_)	DPP IV enzyme inhibitory activity (% inhibition) (*R*_4_)
B	30	45	60				
C	10	35	60				
D	30	45	60				

Solvent ratio (% *v/v*) (A); Solvent–solid ratio (% *v/w*) (B); Temperature (°C) (C); Time (min) (D).

**Table 2:** Quadratic polynomial equations for four responses in terms of coded factors.

Response	Equation
*R*_1_	*R*_1_ = 85.76 + 0.5233 A + 0.1925 B + 7.59 C − 0.4033 D − 2.53 AB − 0.7100 AC − 0.0075 AD − 0.3025 BC − 0.2075 BD − 0.4400 CD + 0.3383 A^2^ + 0.7746 B^2^ − 0.9804 C^2^ − 2.2142 D^2^
*R*_2_	*R*_2_ = 62.38 + 0.9508 A + 9.72 B − 0.6917 C + 2.07 D − 2.38 AB + 6.44 AC + 1.92 AD − 1.43 BC + 0.5350 BD − 2.82 CD − 0.365 8A^2^ + 1.81 B^2^ + 1.09 C^2^ + 0.4654 D^2^
*R*_3_	*R*_3_ = 88.62 + 3.32 A − 2.16 B + 0.2000 C + 6.10 D − 0.8850 AB + 9.55 AC − 1.05 AD + 3.52 BC + 0.2025 BD + 0.7500 CD + 2.58 A^2^ + 1.18 B^2^ + 5.79 C^2^ − 12.96 D^2^
*R*_4_	*R*_4_ = 72.69 − 7.02 A − 3.33 B − 5.78 C − 0.6458 D − 6.14A B − 6.46 AC − 0.9875 AD − 4.76 BC − 5.18 BD − 10.87 CD − 4.29 A^2^ − 0.3296 B^2^ − 4.58 C^2^ − 5.81 D^2^

In these equations, A, B, C, and D are the values of the independent variables, *R*_1_*–R*_4_ is the predicted response: solvent ratio (water–ethanol) (% *v/v*) (A), solvent–solid ratio (% *v/w*) (B), temperature (°C) (C), time (min) (D), Extraction yield (*R*_1_), TFC (*R*_2_), TPC (*R*_3_), DPP IV enzyme inhibitory activity (*R*_4_).

**Table 3:** Design matrices of actual and predicted values of water–ethanol ratio (A), solvent–solid ratio (B), temperature (C), and time (D) of extraction conditions for *M. oleifera* leaves using the BBD design.

Run	Code Level				Response Variable							
	A	B	C	D	*R*_1_		*R*_2_		*R*_3_		*R*_4_	
					Act.	Pred.	Act.	Pred.	Act.	Pred.	Act.	Pred.
1	0	−1	0	−1	86.37	86.30	55.38	55.58	70.46	70.46	76.46	76.44
2	0	+1	−1	0	78.17	78.16	72.44	72.14	90.98	90.88	65.00	65.00
3	+1	0	0	−1	89.77	89.71	59.23	59.43	75.76	76.12	80.92	80.92
4	+1	0	+1	0	92.31	92.00	70.24	72.48	110.48	110.52	79.88	80.28
5	+1	0	−1	0	79.46	79.40	59.43	59.43	90.98	91.08	76.24	75.11
6	0	+1	0	+1	87.86	86.06	79.00	77.66	81.99	80.28	69.05	69.25
7	+1	−1	0	0	89.30	89.88	57.27	57.27	105.18	105.22	90.42	91.22
8	+1	+1	0	0	85.47	87.27	72.14	72.18	91.82	90.82	74.62	75.12
9	−1	0	−1	0	78.51	78.51	72.84	70.24	100.98	100.98	78.57	78.57
10	0	0	0	0	88.63	88.60	59.87	58.89	84.72	82.42	68.65	68.71
11	0	0	−1	+1	77.66	77.36	72.24	70.64	90.42	90.42	56.54	55.89
12	−1	0	0	−1	88.51	88.71	58.08	58.08	76.02	75.86	65.28	65.08
13	0	0	0	0	86.06	85.06	68.55	68.35	100.90	102.00	69.46	69.44
14	−1	+1	0	0	88.16	89.16	75.65	75.95	80.42	80.73	73.02	73.02
15	0	0	0	0	82.60	82.67	58.71	57.81	80.23	80.26	79.95	80.15
16	0	−1	−1	0	78.46	79.04	52.93	53.33	100.42	100.42	86.01	85.89
17	+1	0	0	+1	82.60	82.66	66.19	64.89	80.90	83.10	81.75	81.55
18	0	0	−1	−1	72.71	72.57	58.88	57.81	73.06	70.88	78.57	79.55
19	0	0	+1	+1	94.20	92.87	63.86	63.76	90.42	90.22	64.29	62.20
20	0	+1	+1	0	91.40	91.40	70.24	75.41	100.34	100.34	63.10	63.10
21	−1	0	+1	0	94.20	94.24	57.89	57.81	82.26	80.54	56.35	56.35
22	0	+1	0	−1	87.61	87.61	76.35	75.65	71.10	70.90	61.11	60.71
23	0	−1	+1	0	92.90	91.63	56.46	56.68	95.68	95.58	65.08	65.18
24	0	0	+1	−1	91.01	90.07	61.77	59.97	70.06	71.38	42.86	43.16
25	−1	−1	0	0	81.88	79.80	51.26	51.86	90.24	90.22	64.25	64.29
26	0	−1	0	+1	87.45	88.21	55.89	56.39	80.54	80.54	63.66	64.10
27	−1	0	0	+1	81.37	81.33	57.37	57.27	85.34	85.34	62.16	62.14

Solvent ratio (% *v/v*) (A); solvent–solid ratio (% *v/w*) (B); temperature (°C) (C); time (min) (D); extraction yield (% *w/w*) (*R*_1_); total flavonoid content (mg QE/g extract) (*R*_2_); total phenolic content (mg GAE/g extract) (*R*_3_); DPP IV enzyme inhibitory activity (% inhibition) (*R*_4_).

**Table 4:** ANOVA for the quadratic polynomial models developed for the response variables: extraction yield (*R*_1_) and TFC (*R*_2_) of *M. oleifera*.

Source	*R*_1_					*R*_2_				
	Sum of Squares	df	Mean Squares	*F*-Value	*p*-Value	Sum of Squares	df	Mean Squares	*F*-Value	*p*-Value
Model	739.17	14	52.80	5.69	0.0023	1472.54	14	105.18	6.69	0.0011
A	3.29	1	3.29	0.3544	0.5627	10.85	1	10.85	0.6902	0.4223
B	0.4447	1	0.4447	0.0479	0.8304	1133.55	1	1133.55	72.11	<0.0001
C	690.84	1	690.84	74.49	<0.0001	5.74	1	5.74	0.3652	0.5569
D	1.95	1	1.95	0.2105	0.6546	51.50	1	51.50	3.28	0.0954
AB	25.55	1	25.55	2.76	0.1228	22.66	1	22.66	1.44	0.2531
AC	2.02	1	2.02	0.2174	0.6494	165.89	1	165.89	10.55	0.0070
AD	0.0002	1	0.0002	0.0000	0.9962	14.71	1	14.71	0.9356	0.3525
BC	0.3660	1	0.3660	0.0395	0.8459	8.21	1	8.21	0.5222	0.4838
BD	0.1722	1	0.1722	0.0186	0.8939	1.14	1	1.14	0.0728	0.7918
CD	0.7744	1	0.7744	0.0835	0.7775	31.75	1	31.75	2.02	0.1807
A^2^	0.6105	1	0.6105	0.0658	0.8019	0.7138	1	0.7138	0.0454	0.8348
B^2^	3.20	1	3.20	0.3450	0.5678	17.50	1	17.50	1.11	0.3121
C^2^	5.13	1	5.13	0.5528	0.4715	6.31	1	6.31	0.4016	0.5382
D^2^	0.2446	1	0.2446	0.0264	0.8737	1.16	1	1.16	0.0735	0.7909
Residual	111.29	12	9.27			188.63	12	15.72		
Lack of Fit	92.98	10	9.30	1.02	0.5930	130.80	10	13.08	0.4523	0.8397
Pure Error	18.31	2	9.16			57.84	2	28.92		
Cor total	850.46	26				1661.17	26			
*R* ^2^	0.9691					0.9864				
Adj. *R*^2^	0.9165					0.9540				
Pred. *R*^2^	0.8218					0.8681				
Adequate precision	7.4695					8.9156				

**Table 5:** ANOVA for the quadratic polynomial models developed for the response variables TPC (*R*_3_) and DPP IV enzyme inhibitory activity (*R*_4_) of *M. oleifera*.

Source	*R*_3_					*R*_4_				
	Sum of Squares	df	Mean Squares	*F*-Value	*p*-Value	Sum of Squares	df	Mean Squares	*F*-Value	*p*-Value
Model	2720.14	14	194.30	4.24	0.0083	2664.05	14	190.29	11.81	<0.0001
A	132.40	1	132.40	2.89	0.1511	590.80	1	590.80	36.67	<0.0001
B	55.77	1	55.77	1.22	0.2918	133.20	1	133.20	8.27	0.0140
C	0.4800	1	0.4800	0.0105	0.9202	401.02	1	401.02	24.89	0.0003
D	445.91	1	445.91	9.72	0.0089	5.01	1	5.01	0.3106	0.5875
AB	3.13	1	3.13	0.0683	0.7983	150.92	1	150.92	9.37	0.0099
AC	365.19	1	365.19	7.96	0.0154	167.18	1	167.18	10.38	0.0073
AD	4.37	1	4.37	0.0952	0.7629	3.90	1	3.90	0.2421	0.6316
BC	49.70	1	49.70	1.08	0.3184	90.54	1	90.54	5.62	0.0354
BD	0.1640	1	0.1640	0.0036	0.9533	107.54	1	107.54	6.67	0.0239
CD	2.25	1	2.25	0.0491	0.8284	472.19	1	472.19	29.31	0.0002
A^2^	35.47	1	35.47	0.7732	0.3965	98.14	1	98.14	6.09	0.0296
B^2^	7.39	1	7.39	0.1612	0.6951	0.5793	1	0.5793	0.0360	0.8528
C^2^	178.87	1	178.87	3.90	0.0718	111.96	1	111.96	6.95	0.0217
D^2^	895.80	1	895.80	19.53	0.0008	180.14	1	180.14	11.18	0.0058
Residual	550.42	12	45.87			193.35	12	16.11		
Lack of Fit	314.02	10	31.40	0.2657	0.9396	113.89	10	11.39	0.2866	0.9291
Pure Error	236.40	2	118.20			79.46	2	39.73		
Cor total	3270.56	26				2857.40	26			
*R* ^2^	0.9317					0.9323				
Adj. *R*^2^	0.9054					0.9134				
Pred. *R*^2^	0.7843					0.7079				
Adequate precision	8.3776					15.8775				

**Table 6:** Predicted and actual response values for the verification model.

Set	A	B	C	D	*R*_1_			*R*_2_			*R*_3_			*R*_4_		
					Act. Value	Pred. Value	RSE (%)	Act. Value	Pred. Value	RSE (%)	Act. Value	Pred. Value	RSE (%)	Act. Value	Pred. Value	RSE (%)
1	50:50	300:10	35	45	88.36	89.74	1.54	59.70	58.43	2.17	98.54	98.74	0.20	93.84	93.80	0.04
2	5:95	300:10	25	35	78.15	80.76	3.23	53.88	53.97	0.17	81.82	83.53	2.05	80.25	78.50	2.23
3	25:75	300:10	10	45	80.24	79.48	0.96	56.74	55.82	1.65	103.01	101.07	1.92	80.73	82.31	1.92

**Table 7:** Predicted and actual response values for the optimized extraction parameters.

Parameter No.	Responses	Actual Values (SD)	Predicted Value (SD)	RSE%
1	*R*_1_	87.99 (0.16)	88.10 (0.11)	0.13
2	*R*_2_	56.63 (0.43)	56.61 (0.44)	0.04
3	*R*_3_	97.26 (0.33)	97.16 (0.30)	0.10
4	*R*_4_	93.32 (0.06)	93.38 (0.04)	0.06

Extraction yield (% *w/w*); TFC (mg GAE/g extract); TPC (mg QE/g extract); DPP IV enzyme inhibitory activity (% inhibition).

## Data Availability

The data presented in this study are available upon request from the corresponding author.
